# TD-NMR-Based Determination of the Entrapped Water Yield of Water-in-Oil-in-Water Double Emulsions: Influence of Xanthan Gum Addition

**DOI:** 10.3390/molecules30244680

**Published:** 2025-12-06

**Authors:** Yulin Hu, Ferre Rebry, Paul Van der Meeren

**Affiliations:** Particle and Interfacial Technology Group, Department of Green Chemistry and Technology, Faculty of Bioscience Engineering, Ghent University, Coupure Links 653, B-9000 Gent, Belgium; yulin.hu@ugent.be (Y.H.);

**Keywords:** W/O/W double emulsions, xanthan gum, entrapped water yield, *T*_2_ relaxation, NMR diffusometry

## Abstract

Water-in-oil-in-water (W/O/W) double emulsions (DEs) are considered promising systems for encapsulating, protecting, and delivering hydrophilic compounds. However, their thermodynamic instability limits their practical application. The addition of stabilizers and/or thickeners is a straightforward strategy to improve their stability. However, the high viscosity of DEs complicates the accurate determination of their entrapped water yield (EY), especially when applying techniques based on phase separation. In this study, two TD-NMR-based techniques (*T*_2_ relaxometry, and NMR diffusometry) were compared to analytical photocentrifugation to evaluate their effectiveness in determining the entrapped water yield of DEs formulated with various concentrations (0–0.8 wt%) of xanthan gum (Xan) in the external aqueous (W_2_) phase. For EY determination, analytical photocentrifugation led to overestimated results for DEs containing xanthan, primarily due to the high viscosity, which inhibited the complete separation between the cream and serum layers. In contrast, after optimizing measurement and analysis conditions to minimize interference from water and/or solute exchange between the inner and outer aqueous phases, *T*_2_ relaxometry and NMR diffusometry yielded comparable EY values for all DEs with or without Xan. Hence, these two TD-NMR-based techniques can be considered direct and reliable methods for EY determination in viscous DE system.

## 1. Introduction

Double emulsions (DEs) of the water-in-oil-in-water (W/O/W) type are multiphase systems where the internal water droplets are entrapped within oil droplets, which are further dispersed in an outer water phase. Its unique structure enables a range of potential applications, such as the encapsulation of water-soluble compounds and the formulation of fat-reduced products [1,2,3]. Nonetheless, DEs are thermodynamically unstable and exhibit diverse instability mechanisms due to their structural complexity. These include creaming, Ostwald ripening, flocculation, and coalescence between inner water droplets, between outer oil droplets, and between the inner and outer aqueous phase [2,4]. Additionally, exchange of solutes between the internal (W_1_) and external (W_2_) water phases can occur without film rupture [5].

To evaluate the stability and functionality of W/O/W emulsions, an important feature is to monitor the amount of enclosed water. Hereby, the entrapped water yield (EY), defined as the volume ratio of W_1_ phase that remains encapsulated in the DEs at the moment of interest to the W_1_ volume originally used in the DE formulation, can be used. Many techniques have been reported in the literature, each presenting its own limitations [6]. The most commonly used method is adding a marker in the W_1_ phase and measuring its release into the W_2_ phase. The marker can be an electrolyte detected through conductivity measurements [7], a fluorescent dye identified via fluorescence spectroscopy [8], or a water-soluble compound quantified using a colorimetric method [9,10]. Although widely used, this method has certain limitations that may cause inaccuracy. First, centrifugation is typically used to separate the continuous phase from the dispersed phase prior to quantification. However, the involved mechanical energy may induce aggregation and/or disruption of some droplets, potentially leading to an additional release of entrapped water [11]. As a result, the EY could be underestimated. In addition, the accuracy may be compromised if interactions occur between marker molecules and emulsion components, such as emulsifiers. For instance, O’Regan et al. reported a high variability in measured encapsulation efficiency of the marker methylene blue due to its association with gelatin present in the W_1_ phase of DEs [12]. Additionally, the release of an encapsulated marker does not always precisely reflect the release of entrapped water. Nollet et al. observed water exchange in the presence of an unbalanced osmotic pressure between the W_1_ and W_2_ phases, whereas the encapsulation efficiency of vitamin B12 remained constant [13]. Our previous research also found that encapsulated valine could be released through diffusion across the intermediate oil layer even without the simultaneous release of water [14]. Hence, to enhance the accuracy of this method, both the properties of the encapsulated marker and the composition of the DEs need to be carefully considered. Rheological methods can also be employed to estimate the enclosed water fraction by considering the correlation between the dispersed phase content and the viscosity of emulsions as described by the Mooney equation [15]. The release of enclosed water decreases the total volume of the dispersed phase, thereby leading to a reduction in the emulsion viscosity. However, for accurate EY estimation, it is essential to assume monodisperse droplets in the DEs and to apply a low dispersed phase content and low shear rates to avoid droplet deformation during measurement [16]. In addition to the dispersed phase content, interactions between the droplets also affect the rheological behavior of emulsions. Moreover, the changes in viscosity due to the release of encapsulated water may not directly correspond to the dispersed phase content if the compositions of the W_1_ and W_2_ phases differ [17]. Analytical photocentrifugation is a simple and straightforward technique that enables real-time, in situ monitoring of separation processes by recording near-infrared light extinction profiles with spatial and temporal resolution [18]. Typically, W/O/W emulsions undergo phase separation during centrifugation, resulting in a cream layer (consisting of the W_1_ and oil phases) and a serum layer (corresponding to the W_2_ phase). By measuring the light transmission as a function of sample height throughout centrifugation, the enclosed water volume fraction can be derived, allowing for the calculation of the EY value. However, the presence of residual interstitial water within the cream layer often results in an overestimated result, and its effect needs to be minimized by optimizing the emulsifier type and the centrifugation parameters. Differential scanning calorimetry (DSC) also offers a strategy for directly characterizing the enclosed water content in DEs. Hereby, the inner and outer aqueous phases can be distinguished based on their different crystallization temperatures, as reflected in the thermoanalytical curves recorded during a controlled freezing process [19]. However, the small sample size used in DSC measurements may cause variability between replicates, especially for unstable DEs. Moreover, water exchange between two water phases may still occur even when the outer water phase is frozen, making it more challenging to accurately quantify the enclosed water fraction [11]. In addition, fat crystallization may interfere with the water–ice transformation. Furthermore, the potential of nuclear magnetic resonance (NMR) for properly determining the encapsulated water fraction has been investigated in several studies. Both pulsed field gradient (PFG) NMR diffusometry and *T*_2_-relaxometry have been applied. The former relies on the different diffusion behavior of water in the W_1_ phase (restricted diffusion) and the W_2_ phase (free diffusion), while the latter distinguishes two water phases based on their different relaxation behaviors upon the addition of a paramagnetic agent to the W_2_ phase [20,21]. NMR allows non-destructive testing, but it requires complex data analysis, and high-field NMR spectrometers are particularly expensive. Moreover, both NMR-based methods require careful parameter optimization to minimize the effect of intercompartment water exchange, or the use of appropriate post-measurement analyses to quantify this effect [20].

Incorporating stabilizers and/or thickeners (e.g., polysaccharides) into the W_2_ phase of DEs is a straightforward approach to improve their stability. On one hand, the increased viscosity of the W_2_ phase restricts the mobility of the oil droplets, thereby inhibiting creaming and aggregation [22,23]. On the other hand, the increase in the W_2_ phase viscosity may affect the droplet generation during homogenization. In this respect, a viscosity ratio (dispersed phase to continuous phase) between 0.05 and 5 has been found to favor droplet breakup and the formation of smaller droplets [8]. Nonetheless, the increase in emulsion viscosity also makes it more challenging to accurately determine the entrapped water yield by methods that rely on phase separation between the dispersed and continuous phase, due to the greater difficulty in achieving complete phase separation by centrifugation. For those viscous DEs reported in the literature, a dilution step followed by centrifugation and/or filtration is commonly used to obtain the serum phase of DEs, from which the release of encapsulated compounds is then determined to calculate the encapsulation efficiency [24,25,26]. However, this method is indirect and has some limitations. Whereas the effect of thickeners on DSC determination of the EY of DEs has been reported by Schuch et al., Oppermann et al., and Zhu et al. [22,27,28], the effectiveness of other methods for determining the EY of DEs formulated with thickeners remains unclear and a quantitative benchmarking of these methods under controlled viscosity gradients is currently lacking.

Accordingly, in this study, we produced DEs with a range of viscosities and conducted a quantitative benchmarking of three direct methods (analytical photocentrifugation and NMR-based techniques including *T*_2_ relaxometry and diffusometry) to evaluate their performance in the EY determination of viscous DEs. DEs were prepared using the lipophilic emulsifier PGPR and the nonionic hydrophilic emulsifier Tween 80. Xanthan gum (Xan), a non-surface-active polysaccharide known for its stability under varying conditions of heat, salt, and pH, which is widely used to stabilize emulsions, was incorporated into the DEs as a thickener to achieve a range of viscosities [24,29]. This quantitative benchmarking aims to clarify the limitations of each method and to identify the most appropriate approach for determining the EY of W/O/W emulsion systems under controlled viscosity gradients.

## 2. Results

### 2.1. Droplet Size

**Figure 1:**
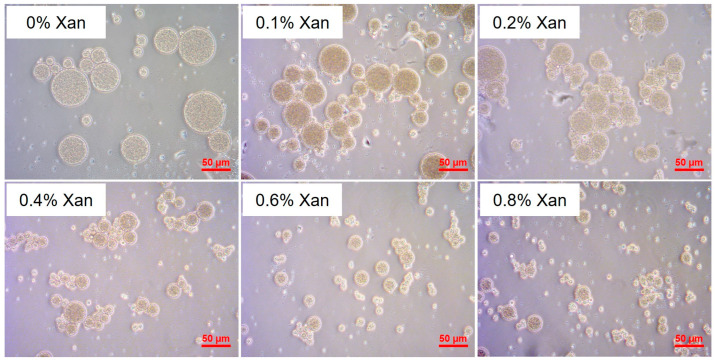
Light microscopic pictures for W/O/W (25/25/50; *w*/*w*/*w*) emulsions formulated with 0–0.8% xanthan (Xan) in the W_2_ phase under 40-fold objective; the scale bar is 50 µm.

All W/O/W DEs were produced successfully, which owned the structure of inner water droplets in oil droplets, as indicated in the light microscopic photos in Figure 1. Obviously, the concentration of Xan in the W_2_ phase showed an effect on the oil droplet size of the DEs.

**Figure 2:**
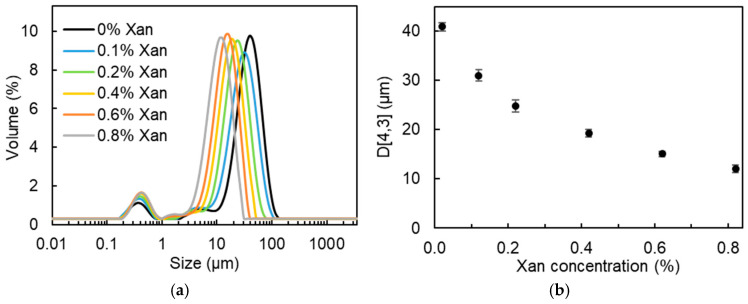
Oil droplet size distribution (**a**) and volume-weighted average oil droplet diameter (D[4,3], (**b**)) for W/O/W (25/25/50; *w*/*w*/*w*) emulsions formulated with 0–0.8% xanthan in the W_2_ phase.

In Figure 2a, all DEs showed a multimodal size distribution; the main peak shifted to smaller droplet sizes with increased xanthan concentration in the W_2_ phase. Hereby, the volume-weighted average diameter (D[4,3]) of the oil droplets decreased from 40.9 µm to 12.0 µm when increasing the Xan concentration in the W_2_ phase from 0% to 0.8% (Figure 2b). This decreased oil droplet size can be explained from the perspective of viscosity. As shown in Figure S1, the viscosity of the W_2_ phase obviously increased with increasing Xan concentration. Moreover, the W_2_ phase containing more than 0.2% Xan showed a pronounced shear-thinning behavior, whereas both the water-in-oil (W/O) emulsions and the W_2_ with 0% and 0.1% Xan displayed nearly Newtonian flow behavior. The viscosity ratio of the primary W/O emulsions to the W_2_ phase, calculated based on the apparent viscosity at a shear rate of 30 s^−1^, was 1235, 131, 23, 3.2, 1.5, and 1.0 for DEs containing 0%, 0.1%, 0.2%, 0.4%, 0.6%, and 0.8% Xan, respectively. Wooster et al. reported that when the viscosity ratio of the dispersed phase to the continuous phase (η_D_/η_C_) approaches a value between 0.05 and 5, the critical Weber number reaches its minimum, favoring droplet break-up [30]. As a result, smaller droplets are generated. A similar effect of Xan concentration on the droplet size of DEs was also reported by Su et al. and Oppermann et al. [22,24].

### 2.2. Analytical Photocentrifugation

**Figure 3:**
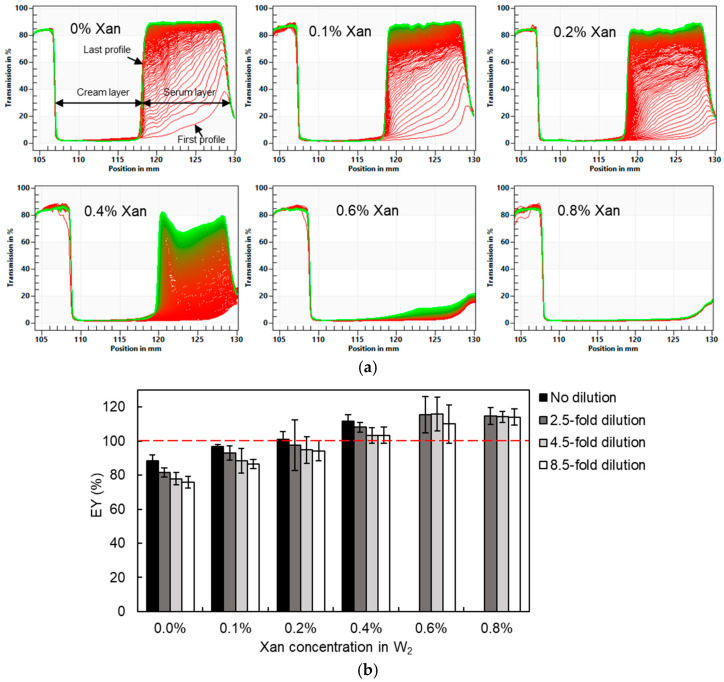
Transmission profiles of undiluted W/O/W emulsions (**a**) and estimated entrapped water yield (EY) for W/O/W emulsions formulated with 0–0.8% xanthan in the W_2_ phase upon different degrees of dilution (**b**). The red dashed line corresponds to an EY value of 100%.

The usefulness of the analytical photocentrifugation technique to determine the entrapped water yield of DEs containing Xan was first verified. To realize the complete separation between the cream layer and the serum layer and to minimize the effect of the interstitial water fraction, the highest centrifugal speed (4000 rpm, corresponding to 2300× *g* of centrifugal force) of the device used was chosen. Figure 3a showed the real-time transmission profiles of DEs during 4 h of centrifugation (720 profiles in total). The early profiles are marked in red and the later profiles in green. After separation, the cream layer with lower transmission and the serum layer with higher transmission can be easily distinguished. It is obvious that the creaming velocity of the DEs was strongly reduced by Xan (from 0% to 0.4%) addition into the W_2_ phase, whereas no separation was observed for samples with 0.6% and 0.8% Xan. Hence, a better stability of DEs against creaming and centrifugation was obtained through adding Xan in the W_2_ phase. However, the higher viscosity and lower droplet size at Xan concentrations above 0.5% inhibited the separation between the cream and serum layers, making it impossible to determine the EY value by centrifugal photosedimentometry. The application of a longer centrifugation duration (up to 16 h) did not produce a noticeable effect on phase separation. In terms of DEs formulated with 0% to 0.4% Xan, their EY values calculated from the transmission profiles showed an increasing trend from 88.6% to 111.5% as the Xan concentration increased (Table 1). An EY value higher than 100% typically indicates water migration from the external water phase into the internal water phase driven by the higher osmotic pressure in the internal phase. However, the osmotic pressure was balanced in our study. Therefore, the higher experimentally determined EY values (even exceeding 100%) are more likely attributable to increased measurement errors associated with higher xanthan concentrations. The incorporation of Xan effectively increases the viscosity of the emulsion system, thereby reducing the creaming velocity of oil droplets in DEs. Meanwhile, Xan facilitates the formation of a weak gel-like network that immobilizes the droplets, which further enhances the resistance to phase separation [31]. In addition, xanthan gum, as a non-adsorbing polysaccharide, can induce depletion-driven droplet flocculation, giving rise to large porous aggregates and dense droplet packing at higher concentrations [32]. Xan also exhibits a strong water-binding capacity, which hinders the removal of interstitial water trapped within the cream layer during centrifugation [33]. Consequently, the measured cream layer height used in Equation (1) (which is assumed to represent the volume of the oil phase and the internal water phase only) becomes overestimated due to incomplete phase separation, which in turn leads to an overestimation of the EY value.

The viscosity of DEs can be reduced by dilution, thereby enabling more efficient separation. The W_2_ phase in the absence of Tween 80 and Xan was used as the diluent to minimize water release driven by osmotic pressure differences. However, some release may still occur due to residual osmotic pressure gradients and the excluded-volume effects associated with polymer molecules. The cream layer was successfully separated from the serum layer after 2.5-, 4.5-, or 8.5-fold dilution, even for DEs containing 0.8% Xan. The estimated EY values of DEs after different degrees of dilution, as determined by analytical photocentrifugation, are shown in Figure 3b. For DEs containing 0–0.4% Xan, the estimated EY values showed a gradual decline with increasing dilution. This effect was most pronounced in DEs without Xan, where a 4.5-fold dilution resulted in a 13% decrease in the estimated yield. As for DEs containing 0.6% and 0.8% Xan, the entrapped water yield could be calculated from the transmission profiles after dilution: they were approximately 115%, irrespective of the dilution degree. The dilution effect indicated that the DEs were unstable upon (isotonic) dilution, especially those with lower Xan concentrations, whereas dilution of DEs with a higher Xan concentration could not eliminate EY overestimation due to residual interstitial water in the cream phase. The instability of the DEs may arise from changes in viscosity, interfacial composition, osmotic equilibrium, and the rate of droplet-droplet collisions after dilution. In addition, it should be noted that due to the reduction in the cream layer fraction in diluted DEs, the uncertainty on the estimated cream layer thickness will increase, causing larger variability between replicates. Hence, the obtained results indicated that analytical photocentrifugation is mainly suitable for concentrated low-viscosity DEs but suffers from incomplete separation between the cream and external water phase at higher viscosities.

### 2.3. T_2_-Relaxometry

#### 2.3.1. Determination of Sensitive Volume

As creaming may occur in DEs, it is necessary to ensure that the whole sample content is located in the sensitive volume range during NMR measurement. The sensitive volume of the NMR instrument used was determined by filling an NMR tube with varying amounts of Milli Q water (i.e., different heights of water) and recording the corresponding integrated peak area in the *T*_2_ distribution. As shown in Figure S2a, the peak area increased linearly with the height of water within the range from 0 mm to 14 mm, whereas the peak area remained constant when more than 14 mm of water was present. Hence, around 0.5 g of sample was added into the NMR tubes to achieve a height of about 10 mm in all measurements. Hereby the obtained response was normalized for the mass.

#### 2.3.2. Effect of MnCl_2_ Concentration

The addition of the paramagnetic agent MnCl_2_ in DEs plays a key role in the signal distinction of the W_1_ and W_2_ phases. Firstly, the effect of MnCl_2_ concentration on the *T_2_* relaxation of the W_2_ phase was investigated. In the absence of MnCl_2_, the signal decayed slowly, corresponding to a relaxation time of about 1.50 s. Increasing the concentration of MnCl_2_ (from 0.01 mM to 10 mM) accelerated the spin-spin (transverse) relaxation process progressively. The relationship between the MnCl_2_ concentration and the *T_2_* relaxation time is shown in Figure S2b. The *T_2_* relaxation time was effectively reduced to 326 ms in the presence 0.1 mM MnCl_2_ and further shortened to 4 ms at 10 mM MnCl_2_.

**Figure 4:**
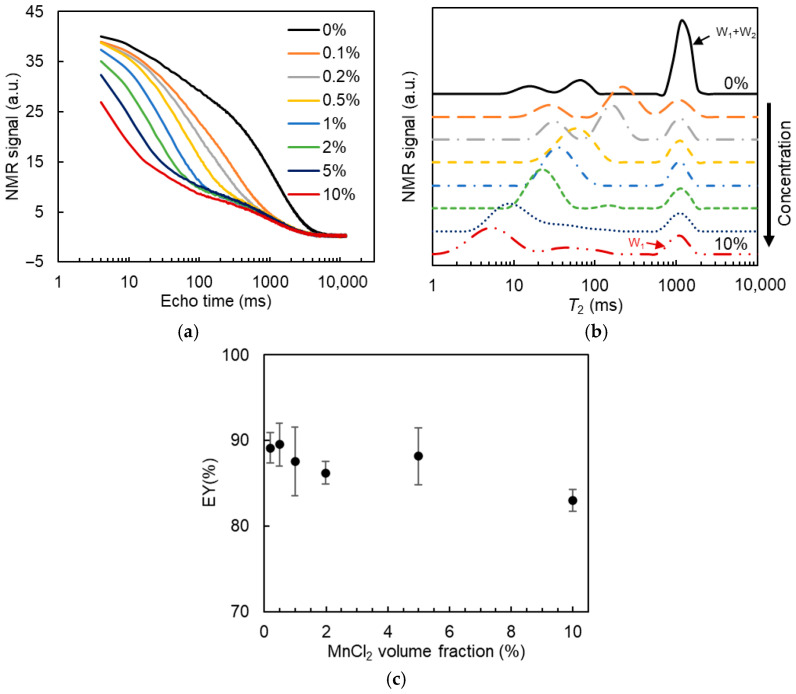
*T*_2_ relaxation decay curve (**a**), *T*_2_ distribution (**b**), and the estimated entrapped water yield (EY, (**c**)) for W/O/W emulsions containing 0% xanthan with the addition of 0–10% (*v*/*v*) 50 mM MnCl_2_ in the W_2_ phase.

Subsequently, the effect of MnCl_2_ concentration on the *T_2_* relaxation of W/O/W DEs was detected. Since the addition of Xan (up to 0.8%) did not show a noticeable effect on the *T*_2_ relaxation distribution of DEs and the signal separation between the W_1_ and W_2_ phases after MnCl_2_ addition (Figure S3), DEs containing 0% Xan were used as a representative system to optimize the parameters of MnCl_2_ concentration and time between MnCl_2_ addition and measurement (data in Section 2.3.2 and Section 2.3.3) in *T*_2_-relaxometry. 50 mM MnCl_2_ was added into DEs at volume fractions of 0.1%, 0.2%, 0.5%, 1%, 2%, 5%, and 10% (*v*/*v*), which resulted in final MnCl_2_ concentrations of 0.1, 0.2, 0.5, 1.0, 1.9, 4.6, and 8.3 mM in the W_2_ phase, assuming no inner water release. All the measurements were performed within 20 min after adding MnCl_2_. Figure 4a illustrates the *T*_2_ relaxation decay curve of the DEs. With increasing MnCl_2_ content in the DEs, the initial signal amplitude decreased, and the signal decay became more rapid, confirming the effect of MnCl_2_ on the *T*_2_ relaxation of the external water phase.

The *T*_2_ distribution was obtained through a regularized inverse Laplace transform. As shown in Figure S3a, the water protons of the W_2_ phase, primary emulsions, and DEs were characterized by a relaxation time of around 1 s, whereas the triglyceride protons of the oil part were characterized by a broad distribution of *T*_2_ relaxation times below 100 ms. Upon the addition of MnCl_2_, the signal from the water protons was clearly separated into two components: a slow-relaxing component at around 1 s and a fast-relaxing component with a relaxation time of less than 0.1 s.

The added amount of MnCl_2_ showed an effect on the *T*_2_ relaxation time distribution of DEs (Figure 4b). At the lowest volume (fraction) of MnCl_2_ solution tested (0.1%), the fast-relaxing signal from the W_2_ phase was not efficiently distinguished from the slow-relaxing signal of the W_1_ phase. At volume fractions of MnCl_2_ solution larger than 0.2%, the two types of relaxation were completely separated, enabling the calculation of the W_1_ phase fraction in DEs. With increasing concentration of MnCl_2_ in the DEs, the fast-relaxing signal gradually shifted to a smaller relaxation time, whereas the distributed signal of the slow relaxation part became broader. The calculated EY values are reported in Figure 4c. A slight decrease in EY value was observed at MnCl_2_ content higher than 1%, while the difference was not statistically significant. When the MnCl_2_ content increased to 10%, the EY value was significantly reduced by around 6%. Combined with the results of the broader distribution of the slow-relaxing part in Figure 4b, it seems that a small amount of MnCl_2_ may have been transported from the W_2_ phase into some inner water droplets, leading to their signal being included in the fast-relaxing water part. Hence, the added amount of MnCl_2_ should be sufficient to ensure clear separation between the inner and outer water phases, but kept to a low value to minimize the effect of MnCl_2_ exchange between the W_1_ and W_2_ phases on the EY results. A volume fraction of (50 mM) MnCl_2_ solution of 0.5% was chosen in our study.

#### 2.3.3. Effect of Time After MnCl_2_ Addition

**Figure 5:**
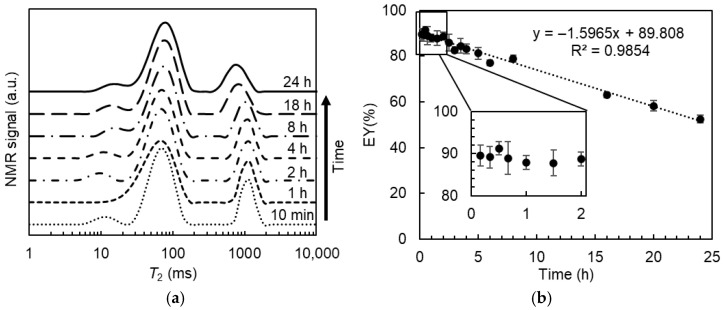
*T*_2_ distribution (**a**) and the estimated entrapped water yield (EY, (**b**)) for W/O/W emulsions containing 0% xanthan measured at different times upon the addition of 0.5% (*v*/*v*) 50 mM MnCl_2_.

Since solute exchange between the W_1_ and W_2_ phases may occur over time, which will affect the accuracy of the calculated EY values, it is essential to optimize the time interval between MnCl_2_ addition and data acquisition. The effect of the time after adding MnCl_2_ on the *T*_2_ relaxation distribution of DEs is reported in Figure 5a. Two types of water (fast- and slow-relaxing) were clearly distinguished from all the relaxation distribution profiles acquired within 24 h after the addition of 0.5% (*v*/*v*) MnCl_2_. For the fast-relaxing part, there was no obvious change over time. For the slow-relaxing part, there was an effect of the time on both the position and the width of the signal distribution. As compared to the results measured after 10 min, the water proton signal of the slow-relaxing water gradually shifted from a relaxation time of around 1.1 s to around 0.7 s after 24 h. Meanwhile, the signal distribution became broader.

The peak areas of the slowly relaxing part were integrated to calculate the encapsulation yield of the DEs. As shown in Figure 5b, the estimated EY values linearly decreased with the storage time upon MnCl_2_ addition. Both the release of enclosed water and the permeability of MnCl_2_ from the outer water phase to the inner water droplets can result in the observed EY decrease. If water release occurs, the MnCl_2_ in the W_2_ phase will be diluted. Consequently, the relaxation time of the fast-relaxing water component is assumed to shift to a larger value, whereas the signal distribution of the slowly relaxing water part will remain unchanged. Therefore, the observed decrease in the estimated EY values can be attributed to the permeability of MnCl_2_ rather than to actual inner water release. In this case, more MnCl_2_ gradually diffuses into the internal water droplets over time, resulting in a shift in their relaxation time towards smaller values. In addition, the distribution of MnCl_2_ in the internal water droplets seemed to be non-uniform, which could be ascribed to their different size and position in the oil droplets. As a result, the proton signal from internal water droplets with more MnCl_2_ permeation may become (partly) included in the fast-relaxing part rather than in the slow-relaxing part, leading to an underestimation of the EY value.

The real EY value can be determined by extrapolating the apparent EY values, plotted as a function of the elapsed time between MnCl_2_ addition and data acquisition, to zero time. The real EY for DEs containing 0% Xan was 89.8% ± 0.46%, consistent with the value (88.6% ± 3.2%) determined from analytical photocentrifugation measurements, confirming the feasibility of determining the entrapped water yield using *T*_2_ relaxometry. Nonetheless, this approach requires more time due to multiple measurements at different time points. The calculated EY values measured within the first 2 h upon MnCl_2_ addition were consistently within the range of 88–91%, which is in good agreement with the real value (89.8%) obtained by extrapolation, and no significant differences were detected. Hence, all subsequent *T*_2_ relaxation measurements were performed within 20 min after adding MnCl_2_, and the acquired data were used to calculate the entrapped water yield of DEs containing different amounts of Xan.

#### 2.3.4. Yield Determination for DEs Containing Xan

With the Xan concentration in the W_2_ phase increased from 0% to 0.8%, the *T*_2_ relaxation time of the W_2_ phase slightly decreased from 1.38 s to 1.08 s (Table S1). Nonetheless, the relaxation signal from the water phase (W_1_ + W_2_) in the corresponding DEs consistently exhibited a monomodal distribution, and the W_1_ and W_2_ phases could always be clearly distinguished after adding 0.5% 50 mM MnCl_2_ (Figure S3). Therefore, this method is effective for EY determination in DEs with Xan concentrations up to 0.8%. The EY values of DEs containing 0–0.8% Xan in the W_2_ phase, as determined by *T*_2_-relaxometry, are presented in Table 1. In the absence of Xan, the entrapped water yield was 87.7%. As the Xan concentration in the W_2_ phase increased from 0.1% to 0.8%, the yield of the corresponding DEs remained within the range of 86–91%. Hence, the addition of Xan did not result in a significant change in EY values, despite of the fact that smaller oil droplets were obtained at higher Xan concentrations.

### 2.4. Diffusometry

**Figure 6:**
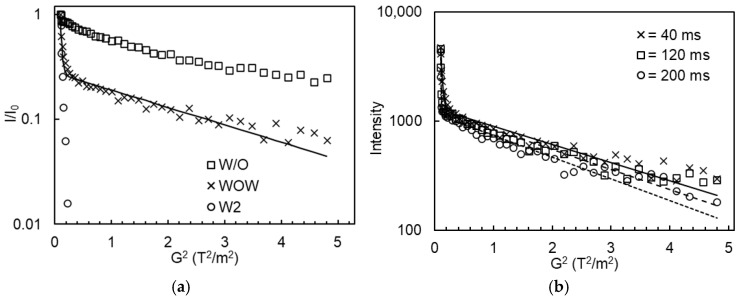
(**a**) The normalized echo intensity of the bulk W_2_ phase, primary W/O emulsions, and W/O/W emulsions containing 0% Xan as a function of the square of gradient strength (G^2^) using a diffusion time of 40 ms; (**b**) The echo intensity of W/O/W emulsions containing 0% Xan as a function of G^2^ using different diffusion times. The markers represent the experimental data, whereas the full and dashed-dotted lines refer to the fitted data using a biexponential function.

PFG-NMR diffusometry allows the distinction between the W_1_ and W_2_ phases in DEs, as illustrated in Figure 6a: the echo intensity of the bulk W_2_ phase showed a rapid exponential decay due to free water diffusion, whereas the W/O emulsions displayed a much slower exponential decay, indicating the restricted water diffusion when entrapped within droplets. As for DEs, a combination of both fast and slow echo decays was observed at low and high G^2^ values, respectively. The data could be well described by a bi-exponential fitting function. Figure 6b shows some typical echo-decay curves of DEs measured at various diffusion times, along with the corresponding bi-exponential model fits. Obviously, the signal attenuation increased with increasing diffusion times, indicating that water exchange occurred between the inner water droplets and the outer water phase during the measurement.

**Figure 7:**
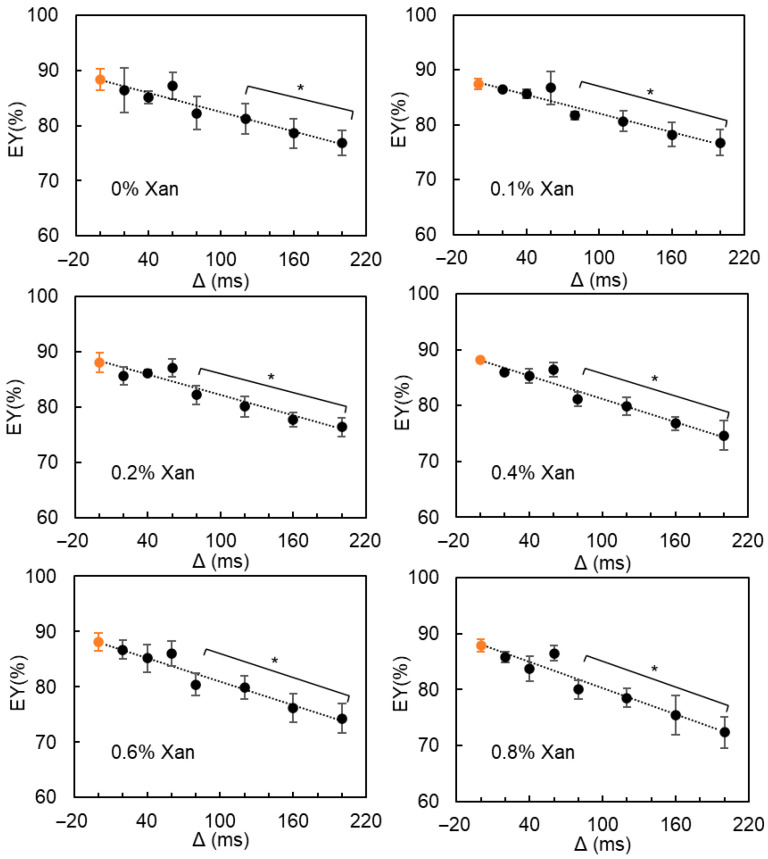
The estimated entrapped water yield (EY) from pfg-NMR diffusometry of W/O/W emulsions containing 0–0.8% Xan in the W_2_ phase as a function of diffusion time (Δ). The orange circular marker represents the EY value obtained by extrapolating the measured EY values to zero diffusion time. * indicates a significant difference between the measured and real EY values.

The water exchange between the W_1_ and W_2_ phases was further confirmed by the estimated EY values as a function of diffusion time (Figure 7): for all DEs (without or with 0.1–0.8% Xan), the estimated EY generally decreased with increasing diffusion time (ranging from 20 ms to 200 ms). As described by Vermeir et al. [20], the real EY values could be approximated by extrapolating the measured EY values to zero diffusion time, which are listed in Table 1. The entrapped water yield of all DEs remained stable at around 88%, despite the formation of smaller oil droplets caused by the addition of more Xan in the W_2_ phase.

## 3. Discussion

The estimated entrapped water yield values of DEs formulated with 0–0.8% Xan in the W_2_ phase, as determined by different methods, were summarized in Table 1 to facilitate comparison. For DEs without xanthan in the W_2_ phase, the EY values determined by analytical photocentrifugation, *T*_2_ relaxometry, and diffusometry were all around 88% and no significant difference was observed. These comparable results from several independent methods indicated that the estimated EY values reflected the real entrapped water yield. Therefore, all these techniques can be considered effective for this low-viscosity system. With the addition of Xan in the W_2_ phase, discrepancies were observed in the estimated EY values of DEs obtained by different methods.

Firstly, analytical photocentrifugation resulted in noticeably different results from the other methods as the estimated EY values gradually increased with increasing Xan concentration in the W_2_ phase. In addition, at Xan concentrations above 0.2%, the estimated EY values exceeded 100%, reaching up to 115%. EY values exceeding 100% might indicate water migration from the W_2_ phase to the W_1_ phase, as is typically caused by a higher osmotic pressure in the W_1_ phase than in the W_2_ phase. However, this was not the case in our study, as the osmotic pressures of the W_1_ and W_2_ phases were basically balanced by the addition of salt. Hence, the EY values determined by analytical photocentrifugation for DEs with higher Xan concentrations were likely overestimated due to measurement errors. This could be ascribed to the fact that the increased viscosity of the continuous phase (and concomitant decreased droplet size), the formation of a weak gel-like network, and the dense packing of oil droplets induced by depletion flocculation hampered the complete separation of the cream layer from the serum layer. Overall, our results clearly indicated that analytical photocentrifugation (even upon moderate dilution) is not suitable for determining the entrapped water yield of viscous DE systems.

Regarding NMR, two independent methods, i.e., *T*_2_ relaxometry and diffusometry, resulted in comparable EY values for all DEs, irrespective of their xanthan concentration and hence viscosity, indicating the reliability of the estimated results. For *T*_2_ relaxometry, MnCl_2_ diffusion from the W_2_ phases to the W_1_ phase might cause an underestimation of the EY values. Hence, it is fundamental to optimize the measurement parameters, including the MnCl_2_ concentration and the time interval between the MnCl_2_ addition and the measurement. Moreover, although the *T*_2_ relaxation time of the W_2_ phase gradually decreased with increasing xanthan concentration, the relaxation signal of the W_1_ and W_2_ phases still overlapped in the *T*_2_ distribution of DEs without MnCl_2_, whereby their complete resolution was obtained upon MnCl_2_ addition, enabling the calculation of the EY using *T*_2_ relaxometry. Overall, *T*_2_ relaxometry proved to be a feasible method for determining the entrapped water yield of viscous DEs formulated with Xan as a thickener. Concerning NMR diffusometry, water diffusion between the W_1_ and W_2_ phases leads to an underestimated EY value for all DEs. The real EY value can be estimated by extrapolating the measured EY values to zero diffusion time. With the addition of Xan (up to 0.8%) in the W_2_ phase, the water fraction of the W_1_ and W_2_ phases could still be reliably estimated via a bi-exponential fitting, despite a slight reduction in the water diffusion coefficient with increasing Xan concentration. Hence, NMR diffusometry was also shown to be effective for EY determination in DEs containing Xan and is particularly recommended when non-destructive testing is required, as it does not require any sample preparation (such as separation of cream and serum layer or MnCl_2_ addition).

## 4. Materials and Methods

### 4.1. Materials

The lipophilic emulsifier polyglycerol polyricinoleate 4110 (PGPR) was generously supplied by Palsgaard A/S (Juelsminde, Denmark). The hydrophilic emulsifier polysorbate 80 (Tween 80) and the thickener xanthan gum from Xanthomonas campestris were procured from Sigma-Aldrich (St. Louis, MO, USA). L-valine (Sigma-Aldrich, St. Louis, MO, USA) was encapsulated in DEs. Sunflower oil (SO) was bought in a local supermarket (Colruyt, Belgium). The water phase contained 107.5 mM potassium chloride (KCl) (VWR Chemicals, Leuven, Belgium) as an osmotic agent and 0.02% sodium azide (NaN_3_) (Sigma-Aldrich, Steinheim, Germany) as an anti-microbial agent. The paramagnetic agent manganese dichloride (99.99%) was acquired from Alfa Aesar (Karlsruhe, Germany). Unless specified differently, the chemicals were of analytical grade.

### 4.2. Preparation of W/O/W Emulsions

The W_1_ and W_2_ phases, and the oil phase were prepared first. The W_1_ phase contained 0.1 M KCl, 0.02 wt% NaN_3_, and 15 mM L-valine. The oil phase consisted of SO with 5 wt% PGPR as the lipophilic emulsifier. The W_2_ phase contained 0.1075 M KCl and 0.02 wt% NaN_3_ to balance the osmotic pressure, 2 wt% Tween 80 as the hydrophilic emulsifier, and xanthan gum at different concentrations (0 wt%, 0.1 wt%, 0.2 wt%, 0.4 wt%, 0.6 wt%, and 0.8 wt%) to generate DEs with varying viscosities.

A two-step process was employed in the preparation of the W/O/W emulsions. Specifically, primary W/O emulsions (50/50, *w*/*w*) were produced via high-shear mixing at 24,000 rpm for 5 min using an Ultra-Turrax homogenizer (S25-10G, IKA-Werke, Staufen, Germany). The corresponding W_1_ phase and oil phase were pre-heated at 60 °C. After cooling to room temperature, the primary W/O emulsions were added into W_2_ phase at a ratio of 50/50 (*w*/*w*) in an ice-water bath. A high shear mixing step at 24,000 rpm for 5 min was used for homogenization to obtain W/O/W DEs (25/25/50, *w*/*w*/*w*). All DEs were stored at 4 °C before analysis.

### 4.3. Light Microscopy

The morphology of DEs was observed using an optical microscope (Olympus Cx40, Tokyo, Japan) equipped with a digital camera (AxioCam ERc 5S, CTK Instruments, Carlsbad, CA, USA) at 40× magnification. Prior to observation, DEs were diluted 10–20 fold with an aqueous solution of 107.5 mM KCl and 0.02 wt% NaN_3_.

### 4.4. Droplet Size

The droplet size distribution and volume-weighted average diameter (D[4,3]) of DEs were determined via static light scattering using a Mastersizer 3000 instrument equipped with a Hydro MV unit (Malvern Instruments, Malvern, UK). Data-analysis was based on the Mie theory, with refractive indices set at 1.33 (continuous phase) and 1.53 (dispersed phase). The dispersion unit was filled with 0.1075 M KCl solution to prevent osmotic effects. The measurement was conducted with constant stirring at 2500 rpm and the obscuration level was maintained between 10% and 20%.

### 4.5. Viscosity

The apparent viscosity of the W/O emulsions and the W_2_ phases containing various concentrations of Xan were measured at 25 °C using a portable viscometer (LV-DVII + PRO, Brookfield, Stoughton, MA, USA) equipped with spindles SC4-18 and SC4-34, a small sample adapter, and an external temperature control unit. The apparent viscosity was recorded as a function of shear rate.

### 4.6. Yield Determination by Analytical Photocentrifugation

According to the method described by Balcaen et al. [18], emulsion samples were centrifuged in rectangular synthetic cells (2 mm path length) at 4000 rpm (2300× *g*) and 25 °C for 4 h using a LUMiSizer (LUM GmbH, Berlin, Germany). Transmission profiles were recorded every 20 s, resulting in a total of 720 profiles. Hereby, the DEs were separated into two layers: a cream layer containing W_1_ and oil, and a serum layer (W_2_), with their start and end positions recorded by front tracking at a transmission of 30%. The entrapped water yield was calculated according to the cream layer height (H_cream_, mm) and the total sample height (H_total_, mm) using Equation (1). H_cream_ and H_total_ represent the cream layer height and the total sample height. V_oil_ and V_W1_ represent the volume fraction of oil phase (i.e., 26.78–26.82% for DEs containing 0–0.8% Xan) and of initial W_1_ phase (i.e., 24.44–24.48% for DEs containing 0–0.8% Xan).

(1) $$EY(\%)=\frac{\left(\frac{H_{cream}}{H_{total}}-V_{oil}\right)}{V_{W_{1}}}\times 100\%$$

### 4.7. Yield Determination by NMR

#### 4.7.1. *T*_2_ Relaxometry

The *T*_2_ relaxation measurements were performed using a Spin Track Time-Domain NMR analyzer (Resonance Systems, Kirchheim unter Teck, Germany) operating at 18 MHz. The magnet system was connected to a sample temperature controller, an external water bath, an air generator, and an air dryer to control the temperature at 5 °C. W/O/W DEs without and with the addition of 50 mM MnCl_2_ were loaded into glass NMR tubes with a diameter of 10 mm; unless otherwise specified, 0.5% *v*/*v* MnCl_2_ was used and 0.5 g of sample was taken. To prevent an osmotic imbalance due to dilution, the ionic strength of the MnCl_2_ solution was adjusted by adding 32.5 mM KCl. The transverse relaxation time (T_2_) was determined using the Carr–Purcell–Meiboom–Gill (CPMG) sequence, consisting of a 90° excitation pulse (2.3 µs) followed by a series of spin echoes (echo delay/2 − 180º refocusing pulse (4.5 µs) − echo delay/2)_n_. A multi-exponential decaying curve is thereby obtained, defined by the time constant *T*_2_. The CPMG sequence was automatically executed using a script in the Relax8 software (version: 8.10.1.1), with 3000 echoes, an echo time of 4 ms, four scans per point, and a relaxation delay of 8000 ms. Each measurement was completed within approximately 3 min. The obtained signal decay curves were converted into *T*_2_ distributions by a regularized inverse Laplace transform, performed via the “rilt” function in MATLAB R2025a with a regularization parameter α = 0.1.

**Figure 8:**
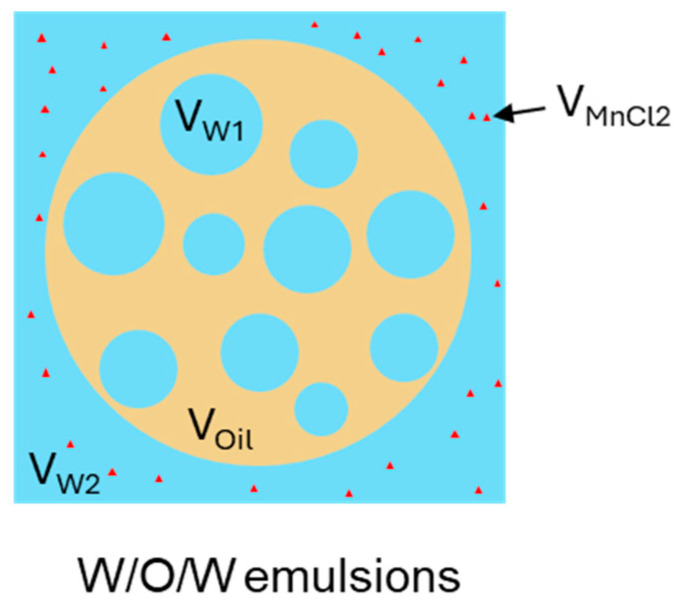
Schematic diagram of different phases (in terms of volume) in W/O/W emulsions in the presence of MnCl_2_.

Figure 8 provides a schematic illustration of the different phases present in W/O/W DEs containing MnCl_2_. The entrapped water yield (EY) was calculated using Equation (2) which consists of four terms. In the first term, A_Ws_ and A_W_ represent the integrated peak areas of the slow relaxation part in the DEs with (W/O/W_m_) and without MnCl_2_ (W/O/W), corresponding to the internal water (W_1_) and the total water (W_1_ + W_2_) in DEs, respectively. The second term accounts for the dilution effect caused by the addition of MnCl_2_ by considering the volume fraction of MnCl_2_ added (V_MnCl2_ = 0.5%, unless otherwise specified) and the total water volume fraction of the DEs (V_W_ = 73.22–73.18% for DEs containing 0–0.8% Xan). In addition, the integrated peak areas are normalized by the mass of W/O/Wm (m_W/O/Wm_) and W/O/W (m_W/O/W_) emulsions in the third term. Finally, the enclosed water fraction in the DEs was determined by scaling with V_W_ and compared with the initial W_1_ volume fraction (V_W1_ = 24.44–24.48% for DEs containing 0–0.8% Xan) to derive the EY.

(2) $$EY(\%)=\frac{A_{W_{s}}\;of\;W/O/W_{m}}{A_{W}\;of\;W/O/W}\times (1+\frac{V_{{MnCl}_{2}}}{V_{W}\;of\;W/O/W})\times \frac{m_{W/O/W}}{m_{W/O/Wm}}\times \frac{V_{W}\;of\;W/O/W}{V_{W_{1}}\;of\;W/O/W}\times 100\%$$

#### 4.7.2. Diffusometry

Pulsed Field Gradient (PFG) NMR diffusometry employs a stimulated echo pulse sequence preceded by an inversion recovery procedure to suppress the oil signal. Meanwhile, a pair of gradient pulses are applied to encode and decode spin positions. The measurements were conducted at 5 °C on a Spin Track Time-Domain NMR analyzer operating at 18 MHz. The gradient strength (G) was varied from 0.01 to 2.17 T/m in 39 steps, whereas the gradient duration (δ) was fixed at 3 ms and the diffusion time (Δ) was set within the range of 40–200 ms. To determine the free self-diffusion coefficient (D) of the different water phases, measurements were performed using varied G ranging from 0.11 to 0.65 T/m, with a δ value of 3 ms and a Δ value of 40 ms. Each measurement was completed within 10 min with a relaxation delay of 8000 ms and one scan per point. The D values were subsequently calculated according to the Stejskal-Tanner equation [21].

*T*_1_-relaxation measurements of the water phases were carried out using an inversion-recovery (IR) sequence with the delay time τ varied from 2 ms to 20 s in 29 steps. For the oil sample, τ was varied from 1 ms to 600 ms, also in 29 steps. A relaxation delay of 8 s was used for the water phases and 1 s for the oil, with 4 scans acquired per point. The *T*_1_ relaxation time was obtained by exponential fitting using the Relax8 software (version: 8.10.1.1). The *T*_1_ values of the different water phases were all larger than 1 s (Table S1), whereas the *T*_1_ of the oil phase was approximately 60 ms.

The optimal time period (τ_0_) used in PFG NMR measurements was determined to be 49 ms, at which the signal intensity of the oil in the PFG-NMR measurement was almost completely suppressed (<50). Hence, at this τ_0_, the oil signal was effectively eliminated while the water signal was preserved due to its significantly longer *T*_1_.

As described by Vermeir et al., the fraction of enclosed water in the total water (EV) can be estimated by fitting a biexponential function to the data of echo attenuation as a function of G^2^ (Equation (3)) [20]. Hereby, γ is the gyromagnetic ratio (2.675 × 10^8^ s^−1^ T^−1^), whereas Di and De represent the diffusion coefficients of water in the internal and external water phases, respectively. The EY was subsequently calculated by Equation (4), whereby V_W1_ and V_W_ represent the total water volume fraction (i.e., 73.22–73.18% for DEs containing 0–0.8% Xan) and the initial W_1_ volume fraction (i.e., 24.44–24.48% for DEs containing 0–0.8% Xan) in the DEs, respectively.

(3) $$\begin{array}{ll} E\left(G^{2}\right)=I/I_{0}= & EV\times exp(-Di\times \Delta \times (\gamma \times \delta \times G{)}^{2})\\ & +\left(1-EV\right)\times exp(-De\times \Delta \times (\gamma \times \delta \times G{)}^{2}) \end{array}$$

(4) $$EY(\%)=\frac{EV\times V_{W}}{V_{W_{1}}}$$

If water exchange between the inner and outer water phases occurs, the estimated internal water fraction tends to decrease with increasing diffusion time due to the less restricted water diffusion in the W_1_ phase. Vermeir et al. suggested that extrapolating the apparent EV values as a function of diffusion time (Δ) to zero Δ provides an approximation of the real EV value [20].

### 4.8. Statistical Analysis

All data are presented as the mean ± standard deviation from a minimum of three replicates. Statistically significant difference (*p* < 0.05) was assessed using one-way analysis of variance (ANOVA) performed in IBM SPSS Statistics 31, followed by Tukey’s post hoc test for multiple comparisons.

## 5. Conclusions

In this study, xanthan gum, a thickener, was incorporated into the W_2_ phase at various concentrations (0–0.8 wt%) to produce double emulsions. A comparative evaluation of three direct techniques (analytical photocentrifugation, *T*_2_ relaxometry, and PFG-NMR diffusometry) was conducted to verify their effectiveness in the EY determination of these viscous DEs. In the case of DEs without Xan, all three methods provided similar results, with an EY value of around 88%. Therefore, these direct methods proved effective for determining the entrapped water yield of low-viscosity DE systems.

The incorporation of Xan into DEs showed an effect on the accuracy of EY results determined by analytical photocentrifugation. In general, the estimated EY values gradually increased with increasing Xan concentration in the W_2_ phase. As the EY determination by analytical photocentrifugation relies on the phase separation via centrifugation, the increased viscosities at higher Xan concentrations hampered the complete separation of the cream layer from the serum layer in DEs, thus resulting in overestimated EY values. For *T*_2_ relaxometry, a longer time (>2 h) between the addition of paramagnetic agent MnCl_2_ and the measurement led to an underestimation of the EY value due to MnCl_2_ exchange between the W_1_ and W_2_ phases, whereas the concentration of MnCl_2_ showed little effect on the estimated EY values. The appropriate EY values for all DEs can be obtained by *T*_2_ relaxometry using the optimized parameters. In addition, PFG-NMR diffusometry, combined with a biexponential fitting, yielded EY values that were comparable with those determined by *T*_2_ relaxometry. Extrapolating the measured EY values as a function of diffusion time to zero diffusion time provided the real EY values of DEs by eliminating the interference from water exchange between the inner and outer water phase. Overall, the two NMR-based techniques, *T*_2_ relaxometry and diffusometry, are recommended for accurate EY determination in viscous DE systems, while methods based on phase separation, even with additional dilution steps, are not suitable.

## Figures and Tables

**Table 1:** Entrapped water yield (EY) of W/O/W emulsions containing 0–0.8% xanthan in the W_2_ phase determined by different methods.

Method	EY (%)					
	0% Xan	0.1% Xan	0.2% Xan	0.4% Xan	0.6% Xan	0.8% Xan
Analytical photocentrifugation (no dilution)	88.6 ± 3.2 ^a^	96.9 ± 0.9 ^a^	100.8 ± 4.9 ^a^	111.5 ± 3.8 ^a^	-	-
Analytical photocentrifugation (4.5-fold dilution)	77.8 ± 3.6 ^b^	88.5 ± 7.2 ^b^	94.7 ± 7.8 ^ab^	103.3 ± 4.5 ^b^	115.9 ± 10.1 ^a^	114.3 ± 3.3 ^a^
*T*_2_ relaxometry	87.7 ± 2.6 ^a^	88.9 ± 1.3 ^b^	90.5 ± 2.1 ^b^	88.7 ± 1.9 ^c^	87.0 ± 1.2 ^b^	86.5 ± 3.9 ^b^
Diffusometry	88.3 ± 1.9 ^a^	87.4 ± 1.0 ^b^	88.1 ± 1.7 ^b^	88.1 ± 0.3 ^c^	88.1 ± 1.6 ^b^	87.9 ± 1.1 ^b^

Values are presented as mean ± standard deviation (*n* ≥ 3). Different letters indicate significant differences between the EY values determined by different methods for the same DEs.

## Data Availability

The data are available on request to the authors.

## Supplementary Materials

The following supporting information can be downloaded at https://www.mdpi.com/article/10.3390/molecules30244680/s1: Figure S1: Apparent viscosity of the primary water-in-oil (W/O) emulsions and the external water (W_2_) phases containing various concentrations of xanthan used for the preparation of double emulsions as a function of shear rates; Figure S2: (a) Integrated peak area in the *T*_2_ distribution as a function of the water height in a 10 mm ID NMR tube; (b) *T*_2_ relaxation time of the W_2_ phase as a function of MnCl_2_ concentration; Figure S3: *T*_2_ distribution of the bulk W_2_ phase, primary W/O emulsion, and W/O/W emulsions prepared with 0% Xan (a) and 0.8% Xan (b), in the absence or presence of MnCl_2_. In accordance with the mass ratio of the W/O/W emulsions (25/25/50), the mass of the W_2_ phase and the W/O emulsions used for measurements corresponded to half of the mass of the W/O/W emulsions; Table S1: Overview of *T*_1_-, as well as *T*_2_-relaxation times, and diffusion coefficients of different water phases.

## Abbreviations

The following abbreviations are used in this manuscript:

W/O/W	Water-in-oil-in-water
DEs	Double emulsions
W1	Internal aqueous phase
W2	External aqueous phase
Xan	Xanthan gum
PFG-NMR	Pulsed field gradient–nuclear magnetic resonance
CPMG	Carr–Purcell–Meiboom–Gill
D[4,3]	Volume-weighted average diameter
