# Supplementary material for: TD-NMR-Based Determination of the Entrapped Water Yield of Water-in-Oil-in-Water Double Emulsions: Influence of Xanthan Gum Addition
Source: Molecules. 2025 Dec 6;30(24):4680. doi: 10.3390/molecules30244680 (PMC12735952; doi:10.3390/molecules30244680)
Supplement: Supplementary file 1 [file molecules-30-04680-s001.zip › molecules-4006905-supplementary.pdf]

Supplementary materials

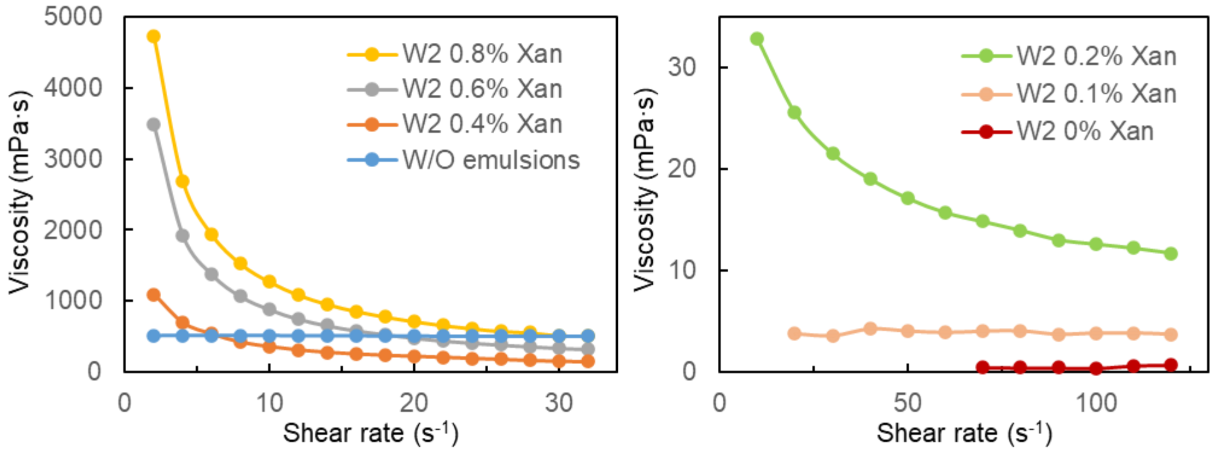

Figure S1. Apparent viscosity of the primary water-in-oil (W/O) emulsions and of the external water (W_2) phases containing various concentrations of xanthan used for the preparation of double emulsions as a function of shear rate.

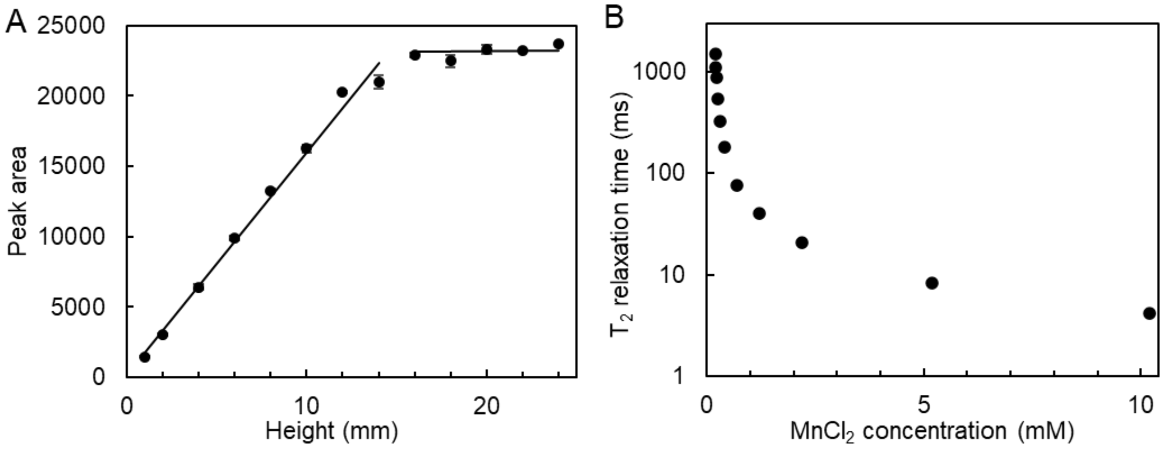

(a) (b)

Figure S2. (a) Integrated peak area in the T_2 distribution as a function of the water height in a 10 mm ID NMR tube; (b) T_2 relaxation time of the W_2 phase as a function of $MnCl_2$ concentration.

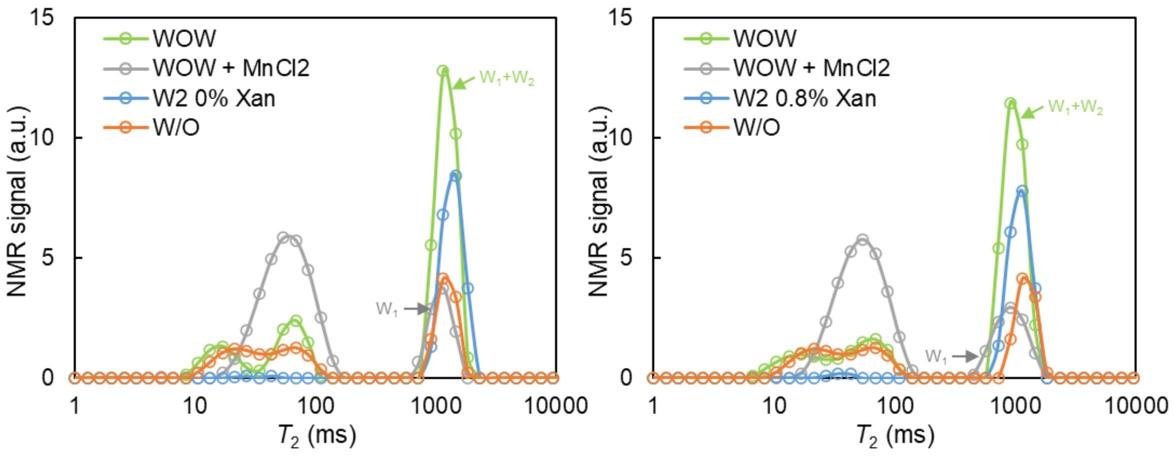

(a) (b)

Figure S3. T_2 distribution of the bulk W_2 phase, primary W/O emulsion, and W/O/W emulsions prepared with 0% Xan (a) and 0.8% Xan (b), in the absence or presence of $MnCl_2$. In accordance with the mass ratio of the W/O/W emulsions (25/25/50), the mass of the W_2 phase and the W/O emulsions used for measurements corresponded to half of the mass of the W/O/W emulsions.

Table S1. Overview of T_1-, as well as T_2-relaxation times, and diffusion coefficients of the internal water phase (W_1; without xanthan) and of the external water phases (W_2; with varying xanthan concentration).

	W_1	W_2					
Xanthan (%)	--	0.0	0.1	0.2	0.4	0.6	0.8
T_2 (s)	1.42±0.00	1.38±0.01	1.36±0.02	1.31±0.00	1.24±0.01	1.19±0.01	1.08±0.03
T_1 (s)	1.49±0.01	1.42±0.00	1.42±0.00	1.42±0.00	1.38±0.00	1.36±0.00	1.35±0.00
D (10^{-9} m^2/s)	1.34±0.09	1.28±0.05	1.26±0.02	1.24±0.04	1.25±0.03	1.23±0.02	1.23±0.02
